# Book Reviews

**Published:** 1904-10

**Authors:** 


Epilepsy and Its Treatment. By William P. Spratling, M.D., Superintendent of the Craig Colony for Epileptics at Sonyea, N. Y. Octavo volume of 522 pages, illustrated. Philadelphia, New York, London: W. B. Saunders & Company. 1904. (Cloth, $4.00 net.)

The first treatise on epilepsy by an American author is likely to attract attention; and when, to that statement, is added the further fact that it embraces the observations made by the writer of the book at the first colony for epileptics established in the western hemisphere, an added interest attaches to its publication at this time.

The treatment of epilepsy for a long time lagged behind the management of other mental disturbances; but, stimulated perhaps by what the Germans are doing for epileptics, Dr. Frederick Peterson and others began to agitate the propriety of establishing a home for the care and treatment of these unfortunates in this state. Mr. Craig, a philanthropist of Rochester, a prominent member of the state board of charities, lent a willing hand and a plethoric purse to the measure, and all these and other influences finally established the colony at Sonyea, which appropriately took the name of Mr. Craig.

Dr. Peterson, with equal appropriateness, became the president of the board of managers and mainly through his solicitation and recommendation, Dr. Spratling was appointed superintendent. This treatise is the recorded experience of the latter during his ten years' incumbency, as well as that gained in the care of epileptics at the New Jersey State Hospital and at the Vanderbilt Clinic. This has undoubtedly offered him the largest experience of any single American observer, hence great value attaches to Dr. Spratling's book.

The volume consists of seventeen chapters besides an introduction and an index, in each and all of which it is addressed to needs of both general practitioner of medicine and undergraduate student. It deals distinctly with the most modern methods of treating epilepsy, and presents them in elaboration without becoming diffuse or wearisome. Indeed, the author is a master of concise and forceful English, therefore presents even his dryest facts in attractive form.

The underlying theme of the treatise, all the way through, is that the individual as well as the disease must be treated. The special forms of mental, moral, physical, and hygienic management, so essential to success and which are made a part of the present day system of treating the epileptic, are set forth with emphasis; in short, Spratling presents a book of today that will be read with interest and profit by every physician.

The manifestations of epilepsy are so peculiar and oftentimes subtle, that even an expert may be puzzled occasionally to affirm with certainty that the disease exists in a given case. This book will serve as an aid in doubtful cases and as a guide to the more intelligent and humane management of a most unfortunate class of afflicted human beings.

Clinical Treatises on the Pathology and Therapy of Disorders of Metabolism and Nutrition. By Prof. Dr. Carl von Noorden, Physician-in-Chief to the City Hospital, Frankfurt a. M. Authorised American edition, translated under the direction of Boardman Reed, M.D., Philadelphia. Part V. Concerning the Effects of Saline Waters on Metabolism. New York: E. B. Treat & Company. 1904. (Price, 75 cents.)

It is important, in these days of recommending patients to "the springs," that intelligent advice should be given. No doubt, saline waters have much influence over digestion; no doubt, also, that they are taken many times without definite reason or systematic method. It becomes physicians to be prepared to give advice in regard to the matter that will at least promise wholesome results; therefore, he should, himself, be well informed concerning balneologic therapy. This book will assist in solving many questions pertaining to the effects of saline waters on the economy.

---

Medical Diagnosis. Special Diagnosis of Internal Medicine. A Handbook for Physicians and Students. By Dr. Wilhelm v. Leube, Professor of Medicine, and Physician-in-Chief to the Julius Hospital at Wurzburg. Authorised Translation from the Sixth German Edition. Edited by Julius L. Salinger, M.D., late Assistant Professor of Clinical Medicine in the Jefferson Medical College. Octavo, pp. 1084. With five colored plates and 74 illustrations. New York and London: D Appleton & Co. 1904. (Price, $5.00.)

The Germans, notoriously close students, are renowned also for accuracy. This is very true with reference to diagnosis. Professor Leube has been known favorably in America for many years, and he is frequently quoted regarding his studies relating to the stomach. None the less is he a diagnostician and this handbook will be welcomed in its American translation by every student and practitioner who is not a master of the German language.

We have had occasion to point out often in these columns, not by any means as an original thought, but as one of the fundamental truths, that diagnosis lies at the threshold of medicine following next to anatomy and physiology. After mastering the latter the neophyte must of necessity turn his attention to the former. In just that proportion to which he attains proficiency in the diagnostic science will his fame as a skilful physician rise. He should stock his library first with works on diagnosis; they are not so numerous but that he should possess himself of each and all. A young physician is to be judged, in large measure, by the nature of the books he keeps company with.

In the very nature of things a work on diagnosis is not fitted to analytical criticism. Leube's distinguishing feature is his accentuation of differential diagnosis, especially in internal diseases. He presents the whole subject in the most modern light. The editor has added some excellent descriptions in brackets, thus bringing the entire treatise forward to the immediate present.

---

Annual Report of the Surgeon-General of the Public Health and Marine Hospital Service of the United States for the Fiscal Year 1903. Washington: Government Printing Office. 1904.

We have called attention heretofore in these columns to the importance of these reports, and to their great interest for all persons bearing official relations to the public health, for teachers of sanitary science, and for those who are pursuing investigation relating to these questions.

The division relating to foreign, insular and domestic quarantine is full of valuable information. The report on domestic quarantine by Assistant Surgeon-General Glennan takes up the subject of the plague at San Francisco, which provoked considerable controversy and generated some ill-feeling on the Pacific Coast two years ago. The propriety of the action taken by the Marine Hospital and Public Health Service cannot be questioned.

The division of scientific research presents some material of interest among which is a report of a case of lipoma arborescens, with photographs and microphotographs, by Assistant T. B. McClintic. The specimen is a rare one and will attract the attention of pathologists.

---

Obstetric and Gynecologic Nursing. By Edward P. Davis, A.M., M.D., Professor of Obstetrics in the Jefferson Medical College and in the Philadelphia Polyclinic. Duodecimo, 402 pages, illustrated. Second edition, revised. Philadelphia, New York, London: W. B. Saunders & Company. 1904. (Polished buckram, $1.75 net.)

Three years ago, when the first edition of this book appeared, it received the commendation of the medical profession as being the most comprehensive as well as the most scientific treatise on obstetric nursing that had been issued. The second edition, now before us, presents the work in revised form with additions, which serves to keep it in the forefront of literature of its class. Every nurse with any pretentions to the name should own this book and make it a part of her regular studies. It is a scholarly treatise, and deserves to rank as a nursing classic.

---

THE PRACTICAL MEDICINE SERIES OF YEAR BOOKS. Ten volumes. Issued under the general editorial charge of GUSTAVUS P. HEAD, M.D., Professor of Laryngology and Rhinology in the Chicago Post-Graduate Medical School. Volume V. Obstetrics. Edited by Joseph B. DeLee, M.D., Professor of Obstetrics, Northwestern University Medical School. Chicago: The Year Book Publishers. 1904. (Price, $1.00; entire series, $5.50, payable in advance.)

This is one of the most important volumes in this excellent series. It exhibits in compact form all the literature of the year pertaining to obstetrics that possesses real value. The compilation is discreetly made by a man of experience in teaching and editing. Considerable space is given, as should be the case, to the pathology of pregnancy and the puerperium. The care of the newborn child, another practical theme, is given important consideration. Altogether, it is a book from which many useful hints in the management of obstetric patients can be obtained, hence is of special value to the general practitioners of medicine.

